# The transcription factors and pathways underpinning male reproductive development in Arabidopsis

**DOI:** 10.3389/fpls.2024.1354418

**Published:** 2024-02-08

**Authors:** Anna Johanna Wiese, Elnura Torutaeva, David Honys

**Affiliations:** ^1^ Laboratory of Pollen Biology, Institute for Experimental Botany of the Czech Academy of Sciences, Prague, Czechia; ^2^ Department of Experimental Plant Biology, Faculty of Science, Charles University, Prague, Czechia

**Keywords:** flower, anther, pollen, reproductive development, transcription factors, pathways

## Abstract

As Arabidopsis flowers mature, specialized cells within the anthers undergo meiosis, leading to the production of haploid microspores that differentiate into mature pollen grains, each containing two sperm cells for double fertilization. During pollination, the pollen grains are dispersed from the anthers to the stigma for subsequent fertilization. Transcriptomic studies have identified a large number of genes expressed over the course of male reproductive development and subsequent functional characterization of some have revealed their involvement in floral meristem establishment, floral organ growth, sporogenesis, meiosis, microsporogenesis, and pollen maturation. These genes encode a plethora of proteins, ranging from transcriptional regulators to enzymes. This review will focus on the regulatory networks that control male reproductive development, starting from flower development and ending with anther dehiscence, with a focus on transcription factors and some of their notable target genes.

## 1 Introduction

Reproductive development in Arabidopsis occurs within a compact flower, where the developmental stage is correlated with floral bud size (Koltunow et al., 1990), making it convenient to study the different stages of this process. Male reproductive development, specifically, occurs within the anthers of stamen, which hold the pollen sacs where pollen grains develop. Male reproductive development is a very complex process involving a multitude of genetic and molecular cues. These developmental events are precisely timed and choreographed. Transcriptional regulation plays a significant role in influencing various aspects of this process. Our understanding of the role that transcription factors (TFs) play in male reproductive development has been greatly assisted by community resources (e.g., TAIR, ABRC, AGI, etc) and male sterile mutants available for Arabidopsis. Unraveling the identity and functions of these TFs has been a central focus of plant reproductive biology research, as it holds the key to understanding the fundamental mechanisms that govern male fertility and the production of seeds. This review will follow along chronologically from flower development through pollen release, discussing the major transcriptional pathways and players involved. Moreover, to keep the review succinct, details about the TFs, their deduced functions, and mutant phenotypes are provided in **Table 1**.

**Table 1:** The transcription factors and downstream target genes involved in male reproductive development.

Gene	Details	Function	Mutant	Phenotypic defects in mutant	Reference
FLOWERING					
*CONSTANS (CO)*	Zinc-finger TF	Regulates flowering under long days	*co-1*	Delayed flowering on long days	Putterill et al., 1995
*FLOWERING D (FD)*	bZIP TF	Interacts with FT to promote flowering	*fd-1*	Late flowering; increased number of rosette leaves	Abe et al., 2005
*AGAMOUS-LIKE 20 (AGL20)* or *SUPPRESSOR OF OVEREXPRESSION (SOC1)*	MADS-box TF	Controls flowering; required for CO to promote flowering	*soc1-2*	Delayed flowering both in long- and short-days growth conditions	Lee et al., 2000
*AGAMOUS-LIKE 24 (AGL24)*	MADS-box TF	Involved in flowering	*agl24-1*	Late flowering in both long- and short-day growth conditions but retains a normal response to photoperiod compared to wild type	Michaels et al., 2003
*LEAFY (LFY)*	HTH TF	Promotes the transition to flowering; involved in floral meristem development	*lfy-1*	Early-arising flowers completely transformed into inflorescence shoots, late-arising flowers partially transformed; petals and stamens absent; sterile	Weigel et al., 1992
*APETALA 1 (AP1)*	MADS-box TF	Specifies floral meristem and sepal identity	*ap1-2*	Homeotic conversion of sepals (first whorl) to bracts (leaf-like structures); secondary or tertiary flowers are formed in the axils of the transformed sepals; petals (second whorl) usually absent but are occasionally replaced by stamens or petal-stamen-bract-like mosaic	Irish and Sussex., 1990
*APETALA 3 (AP3)*	MADS-box TF	Specifies petal and stamen identities	*ap3-1*	Lacks petals: homeotic transformations of petals to sepals, and stamens to carpels; siliques are often misshapen; temperature sensitive	Jack et al., 1992
*AGAMOUS (AG)*	MADS-box TF	Specifies floral meristem and carpel and stamen identity	*ag-1*	Lacks pistils and stamens; homeotic transformation of stamens to petals; a new flower arises in place of the gynoecium; double flowers	Bowman et al., 1991
*PISTILLATA (PI)*	MADS-box TF	Specifies of petal and stamen identities	*pi-1*	Lacks petals and anthers	Goto and Meyerowitz., 1994
*SEPALLATA (SEP)*	MADS-box TF	SEPALLATA proteins act as co-factors with the floral homeotic genes to specify the different floral organs	*sep1 sep2-1 sep3-2*	All flower organs resemble sepals; the second whorl has four green sepal-like organs instead of four white petals; sepals replace stamens in the third whorl; the fourth whorl is a reiteration of whorls 1, 2 and 3.	Pelaz et al., 2000
*SHORT VEGETATIVE PHASE (SVP)*	MADS-box TF	Inhibits flowering	*svp-41*	Recessive mutants have an early flowering phenotype	Hartmann et al., 2000
*WUSCHEL (WUS)*	Homeodomain TF	Keeps the stem cells in an undifferentiated state	*wus-1*	Floral meristems terminate prematurely in a central stamen	Laux et al., 1996
*KNUCKLES (KNU)*	C2H2 type zinc finger TF	Functions as a transcriptional repressor of cellular proliferation that regulates floral determinacy	*knu*	Flowers are conditionally male sterile and contain ectopic stamens and carpels that originate from the placental tissue within the developing gynoecia	Payne et al., 2004
*AINTEGUMENTA (ANT)*	AIL/PLT TF	Regulates growth and cell numbers during organogenesis.	*ant*	Loss of function lines have reduced fertility, abnormal ovules, and abnormal lateral organs.	Klucher et al., 1996
*AINTEGUMENTA-LIKE 6 (AIL6)/PLETHORA 3 (PLT3)*	AIL/PLT TF	Essential for quiescent center specification and stem cell activity.	*plt2-2*	Sensitive to abscisic acid	Yang et al., 2014
*SUPERMAN (SUP)* or *FLORAL DEFECTIVE 10 (FLO10)*	C2H2 type zinc finger TF	Controls the boundary of the stamen and carpel whorls	*flo10*	The fourth whorl is replaced by two to eight stamens or stamenoid-carpels	Schultz et al., 1991
*RABBIT EARS (RBE)*	SUP-like protein with zinc finger motifs	Regulates petal development	*rbe-3*	Defective in sepal, petal, and ovule development; flowers sometimes produce more than four sepals and exhibit fusion between adjacent sepals; petals are often reduced in size or altered in appearance; petals often absent or replaced by staminoid organs and filaments	Krizek et al., 2006
*FLOWERING LOCUS T (FT)*	Florigen, a hormone like molecule	Promotes flowering	*ft*	Late flowering; increased number of cauline leaves	Kobayashi et al., 1999
MICROSPOROGENESIS					
*SPOROCYTELESS (SPL)/NOZZLE (NZZ)*	Putative TF	Involved in sporocyte development	*spl*	Defective in sporocyte formation and anther wall formation; primary sporogenous cells do not form microsporocytes, becoming vacuolated at stage 4; parietal cells are also affected, because the PPCs go through only one division	Yang et al., 1999
*MALE MEIOCYTE DEATH1/DUET (MMD1/DUET)*	PHD-domain containing TF	Regulates microtubule organization and cell cycle transitions during male meiosis	*mmd1*	Male sterile; defect in meiosis II	Reddy et al., 2004
*BRI1 EMS SUPPRESSOR 1 (BES1)*	Brassinosteroid signalling TF	Participates in brassinosteroid signaling; phosphorylated BES1 is destabilized by glycogen synthase kinase-3 (GSK-3) BIN2, a negative regulator of the brassinosteroid pathway	*bes1*	Constitutive brassinosteroid response phenotypes, i.e., long and bending petioles, curly leaves, accelerated senescence, and the constitutive expression of BR-response genes	Yin et al., 2002
*DYSFUNCTIONAL TAPETUM1 (DYT1)*	bHLH TF	Controls anther development and function	*dyt1*	Male sterile; developmental defects in the tapetum at anther stage 4 and later	Zhang et al., 2006
*DEFECTIVE IN TAPETAL DEVELOPMENT AND FUNCTION1 (TDF1)*	R2R3-MYB TF	Regulates tapetal differentiation and function	*tdf1*	Male sterile; defects in anther development; earliest defect seen at developmental stage 6; tapetal cells do not develop into secretory tapetal cells; excessive tapetal cell division; microspores are also developmentally defective	Zhu et al., 2008
*ABORTED MICROSPORES (AMS)*	bHLH TF	Involved in tapetal cell development and the development of microspores into pollen grains	*ams*	Male sterile; premature tapetum and microspore degeneration	Sorensen et al., 2003
*MYB Domain Protein 80 (MYB80)* or *MYB103* or *MALE STERILE 188 (MS188)*	R2R3-MYB TF	Regulates tapetum development, callose dissolution, and exine formation	*ms188-1*	Male sterile; defects in tapetal cell wall degradation and tapetal protoplast degeneration during tapetum development; microspores are either degraded or shrunken and vacuolated; callose dissolution and exine formation are also affected.	Zhang et al., 2007
*MALE STERILITY1 (MS1)*	PHD-finger motif TF	Controls anther and pollen development	*ms1*	Male sterile; pollen degeneration occurs after microspore release; tapetum appears abnormally vacuolated	Wilson et al., 2001
*BARELY ANY MERISTEM1 and 2 (BAM1* and *BAM2)*	CLAVATA1-related receptor kinase-like protein	Important for early anther development, including aspects of cell division and differentiation	*bam1-3 bam2-3*	Male sterile; double mutant anthers lack the endothecium, middle, and tapetum layer; cells interior to the epidermis acquire characteristics of pollen mother cells; pollen mother-like cells degenerate before the completion of meiosis	Hord et al., 2006
*EXCESS MICROSPOROCYTES 1/EXTRA SPOROGENOUS CELLS (EMS1/EXS)*	Putative leucine-rich repeat receptor protein kinase	Controls somatic and reproductive cell fates	*ems1*	Male sterile; excess microsporocytes; lacks tapetal cells; abnormally maintains middle layer cells; failed cytokinesis in microsporocytes	Zhao et al., 2002
*TAPETUM DETERMINANT1 (TPD1)*	Novel small protein similar to proteins of unknown function from other plant species	Involved in cell specification during anther and pollen development	*tpd1-1*	Male sterile; anthers lack tapetal cells and have an increased number of microsporocytes; no tetrads; the middle layer is thicker than in the wild type and remains abnormal	Yang et al., 2003
*SOMATIC EMBRYOGENESIS RECEPTOR-LIKE KINASE1 and 2 (SERK1* and *SERK2)*	Plasma membrane LRR receptor-like serine threonine kinase	SERK1 and SERK2 function redundantly as an important control point for sporophytic development controlling male gametophyte production.	*serk1-1 serk2-1*	Male sterile; in young buds, anthers developed normally, but microsporangia produced more sporogenous cells that were unable to develop beyond meiosis; mutants developed only three cell layers surrounding the sporogenous cell mas; mutant anthers lack development of the tapetal cell layer, which accounts for microspore abortion and male sterility	Colcombet et al., 2005
MICROGAMETOGENESIS					
*Basic Region Leucine Zipper 34 (bZIP34)*	bZIP TF	Involved in exine patterning and the regulation of certain metabolic pathways	*bzip34*	Lipid inclusions in the cytoplam; defects in exine shape and micropatterning; reduced endomembrane system	Gibalova et al., 2009
*NOVEL MICROGAMETOPHYTE DEFECTIVE MUTANT 1 (NMDM1)*	Nuclear protein similar to a transcriptional co-activator	Affects cellulose distribution in the intine by regulating intine-related gene expression	*nmdm1*	Reduced fertility; abnormal pollen intine development	Mi et al., 2022
*MYB Domain Protein 2 (MYB2)*	R2R3 MYB TF	Plays a role in tapetal programmed cell death and pollen developing by activating protease expression	*myb2*	Shrunken pollen with a deformed surface lacking the reticulate ornamentation	Guo et al., 2022
*MYB Domain Protein 81 (MYB81)*	GAMYB TF	Promotes pollen mitosis I and cell lineage formation	*myb81-1*	Male sterile; microspores fail to undergo pollen mitosis I and arrest at the polarized stage with a single central vacuole	Oh et al., 2020
*BONOBO 1 and 2 (BNB1 and BNB2)*	bHLH TFs	Redundantly determine the fate of the generative cell after asymmetric microspore division	*bnb1 bnb2*	Male sterile; generative cells fail to differentiate into the two sperm cells	Yamaoka et al., 2018
*DEFECTIVE REGION OF POLLEN 1 (DROP1) and DROP2*	bHLH TFs	Essential for PMII during generative cell division, ensuring the proper formation of the sperm cells	*drop1 drop2*	Pollen grains are capable of germinating; pollen tubes lack sperm cells; pollen tubes can grow and respond to female signals during the early stages of the journey toward the ovule, and can rupture after entering the ovule.	Zhang et al., 2017
*DUO POLLEN 1 (DUO1)*	R2R3 MYB TF	Required for male gamete formation, specifically for entry of the generative cell into mitosis	*duo1-2*	Male sterile; Defect in sperm cell formation. Generative cell does not undergo mitosis. Produces a single larger diploid sperm cell that is unable to perform fertilization	Rotman et al., 2005
*DUO1-ACTIVATED ZINC FINGER PROTEIN 1 (DAZ1) and DAZ2*	Zinc-finger proteins	Required for germ cell division and for the proper accumulation of mitotic cyclins	*daz1 daz2*	Pollen grains with a single germ cell–like nucleus similar to mutant *duo1* pollen	Borg et al., 2014
*MALE STERILITY2 (MS2)*	Fatty acid reductase	Plays a role in the formation of pollen wall substances	*ms2*	Male sterile; microspore wall development does not occur; no exine layer is formed	Aarts et al., 1993
*POLYKETIDE SYNTHASE A (PKSA)* and *PKSB* or *LESS ADHESIVE POLLEN 6 (LAP6)* and *LAP5*	Plant type III polyketide synthases (PKSs)	Play an role in sporopollenin biosynthesis and exine formation	*pksa pksb*	Male sterile; no apparent exine	Kim et al., 2010
*ACYL-CoA SYNTHETASE5 (ACOS5)*	Fatty acyl-CoA synthetase	Plays an role in sporopollenin biosynthesis and exine formation	*acos5*	Male sterile; mutants devoid of pollen grains at anther maturity; pollen development arrested after their release from tetrads; free microspores are devoid of exine layer	de Azevedo Souza et al., 2009
*TETRAKETIDE α-PYRONE REDUCTASE1 (TKPR1)* or *DIHYDROFLAVONOL 4-REDUCTASE-LIKE1 (DRL1)*	Dihydroflavonol 4-reductase-like1	Plays a role in pollen wall development	*drl1-2*	Male sterile; degeneration of microspores after tetrad release and complete absence of pollen in mature anthers	Tang et al., 2009
*CYP703A2*	Cytochrome P450 family protein	Play an role in sporopollenin biosynthesis and exine formation	*cyp703a2*	Partially male sterile; impaired pollen wall development with absence of exine	Morant et al., 2007
*AUXIN RESPONSE FACTOR 6 (ARF6)*	Auxin response factor	Mediates auxin response via expression of auxin regulated genes. Acts redundantly with ARF8 to control stamen elongation and flower maturation.	*arf6-2*	Short stamens, reduced fertility	Nagpal et al., 2005
*IMPERFECTIVE EXINE FORMATION (IEF)*	Plasma membrane protein	Involved in an independent sporopollenin transportation pathway; essential for exine formation.	*ief-1*	Male sterile; abnormal pollen/microspore development; exine structure not well defined	Wang et al., 2021
ANTHER DEHISCENCE					
*MALE STERILE35 (MS35)*	Putative TF	Regulates secondary wall thickening in the endothecium and is essential for anther dehiscence	*ms35*	Plants are sterile because anthers do not dehisce; the cellulosic secondary wall thickenings are not formed in the endothecium	Yang et al., 2007
*NAC SECONDARYWALL THICKENING PROMOTING FACTOR1* (*NST1*) and *NST2*	NAC TFs	Regulates secondary wall thickening in the endothecium and is essential for anther dehiscence	*nst1 nst2*	Plants are sterile because anthers do not dehisce	Mitsuda et al., 2005
*MYB Domain Protein 21 (MYB21)* and *MYB24*	R2R3 MYB TF	Involved in the jasmonate response during stamen development; function redundantly to regulate stamen development.	*myb21-1*	Reduced male fertility; shorter anther filaments; delayed anther dehiscence	Mandaokar and Browse, 2009

## 2 Floral meristem establishment and floral organ growth

The process of *Arabidopsis* flower development is a thoroughly researched field with a plethora of studies published on the topic. The following section serves as a brief overview of this process, so as to orient the reader for the sections to come.

**Figure 1:**
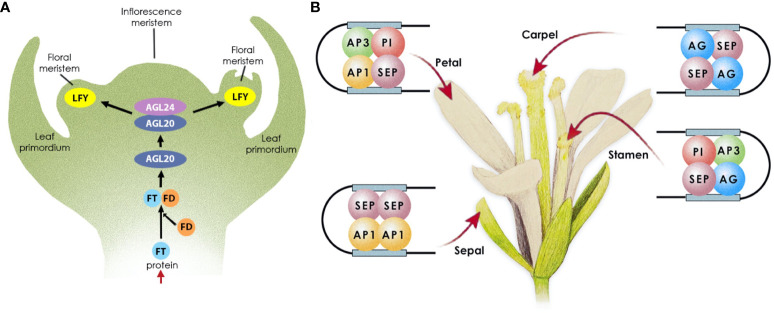
Transcriptional control of flowering in *Arabidopsis*. **(A)** Floral meristem establishment. Following FT expression in leaves, the protein is transported through the phloem to the apical meristem, where it complexes with FD, to activate the expression of *AGL20*; AGL20 then complexes with AGL24 to trigger *LFY* expression in the floral meristem. **(B)** Floral organ growth. LFY initiates the expression of the floral homeotic genes, MADS-box TFs, which subsequently form complexes to promote floral organ growth as follows: AP1 and SEP specify sepals in the outermost whorl; AP1, AP3, PI, and SEP specify petal identity in the second whorl; AP3, PI, AG, and SEP specify stamen identity in the third floral whorl; AG and SEP specify carpel identity in the fourth whorl.

*Arabidopsis* flower development typically follows a precise sequence of events, beginning with the initiation of floral meristems followed by the differentiation of the floral organs (**Figure 1**). As *Arabidopsis* enters the reproductive phase, the meristem responsible for vegetative growth transforms into an indeterminate inflorescence meristem that generates floral meristems on its sides. For this to happen, photoreceptors in the leaves determine the length of the day, and when the day length exceeds a critical threshold, flowering is promoted (Srikanth and Schmid, 2011). However, other cues and environmental factors can also promote flowering. Long-day-dependent flowering initiates the photoperiodic pathway, via CONSTANS (CO) (Putterill et al., 1995), which in turn upregulates the expression of FLOWERING LOCUS T (FT) (Yoo et al., 2005), encoding a mobile protein known as florigen (Kardailsky et al., 1999). The latter is transported from the leaves to the shoot apical meristem (SAM) (Corbesier et al., 2007) where it complexes with FLOWERING D (FD) (Abe et al., 2005). This complex in turn activates the expression of AGAMOUS-LIKE 20 (AGL20) in the inflorescence meristem, directing the shift from vegetative growth to reproductive development (Borner et al., 2000). AGL20 then, in combination with AGL24, activates the expression of *LEAFY* (*LFY*) (Lee et al., 2008) in the SAM. *LFY* then initiates a transcriptional cascade of homeotic genes (*APETALA 1* [*AP1*], *AP3*, AGAMOUS [*AG*], *PISTILLATA* [*PI*], *SEPALLATA1–4* [*SEP1–4*]) that control floral meristem formation and floral organ growth (Kinoshita and Richter, 2020).

LFY and AP1 both function in promoting the expression of the floral homeotic genes required for floral organ development, while concurrently repressing the floral repressor and shoot identity genes, which prevent flowering under unfavourable conditions. Once activated, AP1 and its paralog CAULIFLOWER (CAL) redundantly control the onset of flower development (John L. Bowman et al., 1993; Ferrándiz et al., 2000), by repressing the expression of *SHORT VEGETATIVE PHASE* (*SVP*), *AGL24*, and *AGL20* (Yu et al., 2004; Liu et al., 2007; Liu et al., 2008), the protein products of which repress the activity of SEP3. SEP3 and LFY promote flower development by activating the homeotic floral organ identity genes (Honma and Goto, 2001; Castillejo et al., 2005; Immink et al., 2009). Therefore, *AP1* activation derepresses the expression of *SEP3* to promote flower development. This cascade controls the precise timing of the early events that establish the floral meristem. AG, in turn, promotes floral meristem development by restricting stem cell proliferation (Bowman et al., 1989; Yanofsky et al., 1990). It does so by activating the expression of *KNUCKLES* (*KNU*), which negatively regulates the expression of *WUSCHEL* (*WUS*) (Liu et al., 2011), effectively terminating the floral meristem (Sun et al., 2009).

Once the floral meristem is established, the different floral organs emerge in whorls around the meristem’s sides, from the outermost to the innermost as follows: four green sepals, four white petals, six stamens, and a single pistil. The homeotic genes activated by LFY specify floral organ identity. Their protein products form tetrameric complexes (Honma and Goto, 2001) that specify the different floral organs (Theißen and Saedler, 2001) as follows: AP1 and SEP specify sepals in the outermost whorl; AP1, AP3, PI, and SEP specify petal identity in the second whorl; AP3, PI, AG, and SEP specify stamen identity in the third floral whorl; AG and SEP specify carpel identity in the fourth whorl.

Recently, a novel set of TFs were identified that regulate aspects of floral organogenesis, namely AINTEGUMENTA (ANT) and AINTEGUMENTA-LIKE 6 (AIL6)/PLETHORA 3 (PLT3) (Krizek et al., 2020). ANT directly influences approximately 200 genes involved in auxin signaling, floral organ identity, polarity establishment, growth regulation, and cell differentiation, including AP1, AP2, and SEP3.

For floral organ development to proceed correctly, boundaries (Breuil-Broyer et al., 2004; Reddy et al., 2004) need to be established to restrict the expression of the homeotic genes to their respective whorls. Several boundary genes have been characterized, including *SUPERMAN* (*SUP*) and the closely related *RABBIT EARS* (*RBE*), which repress growth at the boundaries between the third and fourth whorls through *AP3* and *PI* repression (Sakai et al., 1995) and the second whorl through *AG* repression (Krizek et al., 2006), respectively.

## 3 Microsporogenesis

**Figure 2:**
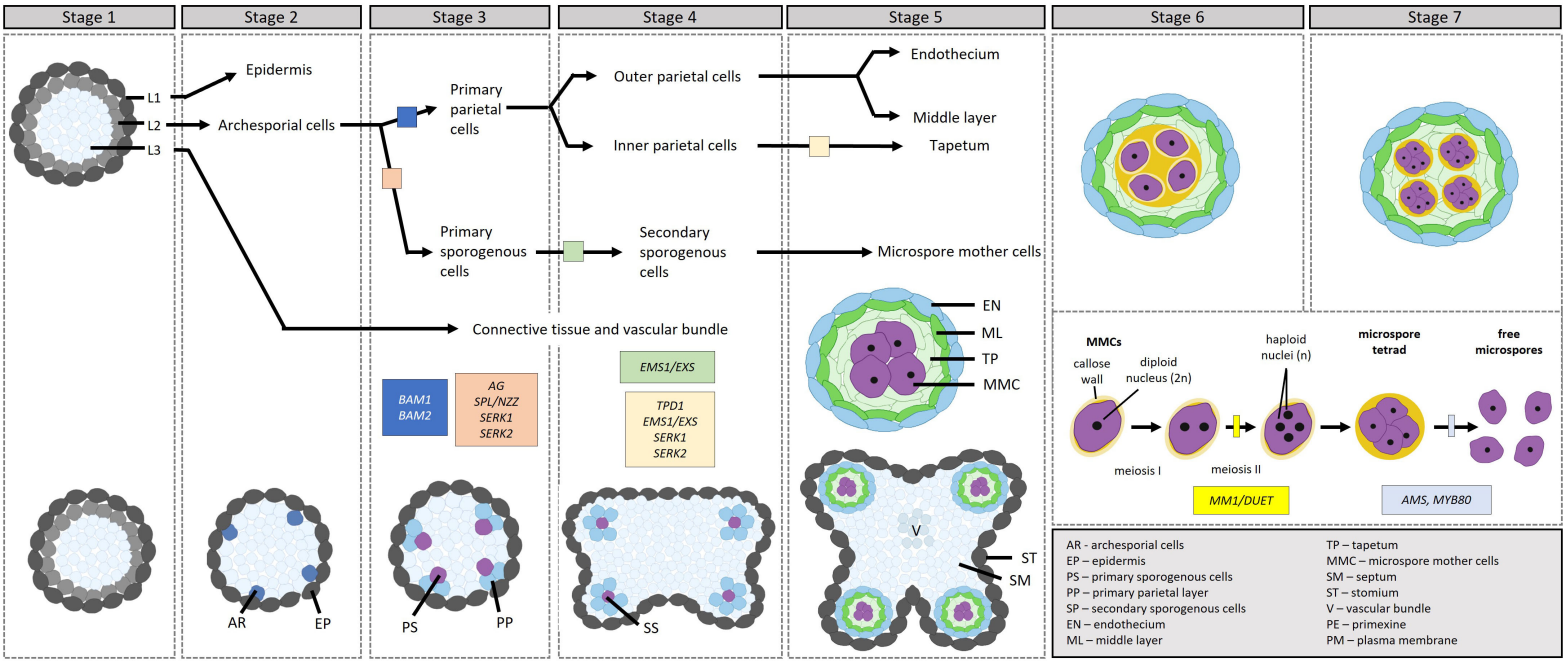
Transcriptional control of microsporogenesis in *Arabidopsis*, development through stages 1–7. [Stage 1] Stamen primordia differentiate into three cell types, L1, L2 and L3. [Stage 2] The cell types further differentiate into the epidermis, the archesporial cells and vascular and connective tissues. [Stage 3] Archesporial cells divide to form the reproductive primary sporogenous cells and the somatic primary parietal layer. [Stage 4] The primary sporogenous cells differentiate into secondary sporogenous cells through the action of EMS1/EXS, while the primary parietal layer differentiates into the outer and inner secondary parietal layers. [Stage 5] The outer secondary parietal layer differentiates into the endothecium and middle layer, while the inner secondary parietal layer differentiates into the tapetum. In addition, the secondary sporogenous cells differentiate into microspore mother cells. [Stages 6–7] The tapetum differentiates further. Microspore mother cells enter and complete meiosis during stages 6 and 7 to produce four microspores in tetrads surrounded by a callose wall. Before the end of stage 7, callose dissollution releases the microspores from the tetrads. The transcription factors involved in the different steps are indicated in the figure in boxes.

Following stamen specification in the third whorl, the stamen primordia undergo further differentiation to give rise to the filament and the anther. The differentiation of the anther in Arabidopsis comprises 14 stages divided into two phases (Sanders et al., 1999; Ma, 2005): microsporogenesis (stages 1–7) and microgametogenesis (stages 8–14). **Figure 2** outlines the process of microsporogenesis, as discussed below. During stage 1, the anther primordium emerges from the floral meristem in the third whorl, consisting of three germ layers (L1–L3). During stages 2–5, the cells in the L1 layer give rise to the epidermis via anticlinal cell division, the cells in the L3 layer differentiate to form vascular and connective tissues, and the cells in the L2 hypodermal layer undergo periclinal cell division to form four clusters of archesporial cells. During stage 3, the archesporial cells in the L2 layer divide to form the reproductive primary sporogenous cells and the somatic primary parietal layer. The primary parietal layer then divides to give rise to two secondary parietal layers; the outer parietal layer further differentiates during stage 4 into the endothecium and middle layers while the cells of the inner parietal layer develop into the tapetal layer. However, recent evidence suggests that the middle layer originates from both the inner secondary and outer parietal cell layers (Zhao et al., 2002; Xue et al., 2021; Feng and Dickinson 2010).

During these early stages of anther development, AG initiates the expression of SPOROCYTELESS/NOZZLE (SPL/NZZ), which is expressed in the L2 layer and plays a role in the specification of sporogenous and primary parietal cells; in the *spl/nzz* mutants, archesporial cells fail to undergo subsequent cell divisions, resulting in sporogenous and primary parietal cells being absent (Schiefthaler et al., 1999; Yang et al., 1999). SPL/NZZ in turn regulates BARELY ANY MERISTEM1 (BAM1) and BAM2, receptor-like kinases (RLKs), which redundantly regulate the formation of the somatic layers (Hord et al., 2006). In the *bam1bam2* mutant, the somatic layers are absent while microspore mother cell (MMC)-like cells are present; the latter, however, degenerate before meiosis. SPL/NZZ and BAM1/BAM2 form part of a feedback loop, wherein SPL/NZZ positively regulates BAM1/BAM2, while BAM1/BAM2 restricts SPL/NZZ’s expression to sporogenous cells, ensuring that SPL/NZZ maintains sporogenous activity and BAM1/BAM2 promotes the development of the somatic cell layers. From here, the EXCESS MICROSPOROCYTES 1/EXTRA SPOROGENOUS CELLS (EMS1/EXS), TAPETUM DETERMINANT1 (TPD1), and SOMATIC EMBRYOGENESIS RECEPTOR-LIKE KINASE1 and 2 (SERK1/2) module specifies the tapetal cell layer (Canales et al., 2002; Zhao et al., 2002; Yang S. et al., 2003). The *ems1/exs*, *tpd1*, and *serk1serk2* mutants are very similar; the L2-derived cell layers are absent, with only the endothecium and middle layers present. In addition, these mutants generate a greater number of MMCs, which can enter meiosis and complete karyokineses, but not cytokinesis, leading to their degeneration. EMS1/EXS and SERK1/2 encode leucine-rich repeat receptor-like kinases (LRR-RLKs) while TPD1 encodes a small protein with a putative signal peptide for secretion. The current model (Ma, 2005) follows: In the differentiating MMCs, the level of EMS1/EXS drops while the level of TPD1 rises; TPD1 is subsequently secreted from the cells and binds to the EMS1/EXS-SERK1/2 complex on the surface of the cells surrounding the MMCs, activating a cascade in the surrounding cells to promote tapetum differentiation. Also downstream of SPL/NZZ, lies the basic region leucine zipper (bZIP) TFs TGA9 and TGA10. These two TFs are expressed in the middle layer and tapetum during stages 5 and 6; mutants show a reduction in tapetum-expressed genes (DYSFUNCTIONAL TAPETUM1 [DYT1] and MALE STERILITY1 [MS1]), affecting microspore and pollen development (Murmu et al., 2010).

By stage 5, all of the somatic cell layers surrounding the developing MMCs are established and the MMCs and tapetal cells are connected via plasmodesmata. Prior to the completion of stage 5, a transient callose (β-1, 3-glucan) layer is synthesized around the MMCs, separating them from each other and the tapetum and breaking the plasmodesmatal connections. Concomitantly, the middle layer thins out. During stage 6, the MMCs enter and complete meiosis to produce four microspores in a tetrad by stage 7, surrounded by a callose wall. Here, MALE MEIOCYTE DEATH1/DUET (MMD1/DUET) is involved in orchestrating the formation of the cell plate, which eventually becomes the cell wall that separates the two daughter cells during meiotic cytokinesis (Yang X. et al., 2003). The accurate positioning and timing of cell plate formation are essential for the successful completion of meiosis.

Whilst encased in a callose layer, following the completion of cytokinesis, the microspores switch from callose to primexine deposition, initiating exine development. The primexine is deposited between the microspore plasma membrane and the callose wall and is largely composed of cellulose, serving as the foundation for sporopollenin precursor accumulation and polymerization on the microspore surface (Fitzgerald and Knox, 1995; Paxson-Sowders et al., 1997; Blackmore et al., 2007). Concurrently, the plasma membrane starts to invaginate, forming undulations, whereon probacula and protectum structures are formed via sporopollenin deposition (Paxson-Sowders et al., 1997).

While the MMCs undergo meiosis, the tapetum further expands and undergoes specialization. Post-specification, the development of the tapetum is controlled by the DYSFUNCTIONAL TAPETUM1 (DYT1), DEFECTIVE IN TAPETAL DEVELOPMENT AND FUNCTION1 (TDF1), ABORTED MICROSPORES (AMS), MYB80, and MALE STERILITY1 (MS1) module, which regulates genes involved in tapetum programmed cell death (PCD) and degradation and pollen wall development (Yang C. et al., 2007a; Zhu et al., 2008; Phan et al., 2011; Gu et al., 2014; Xu J. et al., 2014; Verma, 2019). It was recently shown that a disruption of fatty acid export-mediated ROS homeostasis suppresses the activation of the DYT1-TDF1-AMS-MYB80-MS1 module, resulting in male sterility (Zhu et al., 2020). In this pathway, the master regulator DYT1 lies downstream of EMS1/EXS-TPD1-SERK1/2 pathway. Briefly, the latter pathway mediates the phosphorylation and nuclear localization of BRI1 EMS SUPPRESSOR 1 (BES1), a TF activated by brassinosteroid (BR) signaling. BRs are known to exert a significant influence on pollen development, with BR-deficient mutants having reduced pollen numbers stemming from decreased microspore mother cells and the early abortion of some microspores (Ye et al., 2010). Once in the nucleus, BES1 activates the expression of DYT1 (Chen et al., 2019); BES1 has also been shown to bind the promoters of SPL/NZZ, TDF1, AMS, MS1, and MS2 (Ye et al., 2010). *dyt1* mutants have similar phenotypic defects to that of *ems1/exs* (arrested meiosis progression with no cytokinesis (Zhang et al., 2006). DYT1 in turn activates the expression of TDF1, which is necessary for tapetal cell differentiation and function (Zhu et al., 2008). TDF1 is necessary for the transition of the tapetum to the secretory type, which provides nutrients (e.g., carbohydrates, lipids, proteins, etc) to developing microspores to support their growth and maturation. The *tdf1* mutant shows defective tapetal function and subsequent pollen abortion. TDF1 activates the expression of *AMS* (Lou et al., 2018), which has a multi-pronged effect on tapetum and pollen development (Ferguson et al., 2016). First, by regulating the expression of *TRANSPOSABLE ELEMENT SILENCING VIA AT-HOOK* (*TEK*) in the tapetum, encoding an AT-hook nuclear matrix attachment region (MAR) binding protein (Lou et al., 2014), AMS promotes nexine formation in individual microspores of tetrads following meiosis. This takes place through TEK’s positive regulation of Arabinogalactan protein (AGP) genes (i.e., *AGP6*, *AGP11*, *AGP23*, and *AGP40*), which promote exine formation (Jia et al., 2015). Concomitantly, TEK negatively regulates the expression of *CALLOSE SYNTHASE 5* (*CalS5*) after the tetrad stage, a protein required for callose synthesis (Xiong et al., 2020).

**Figure 3:**
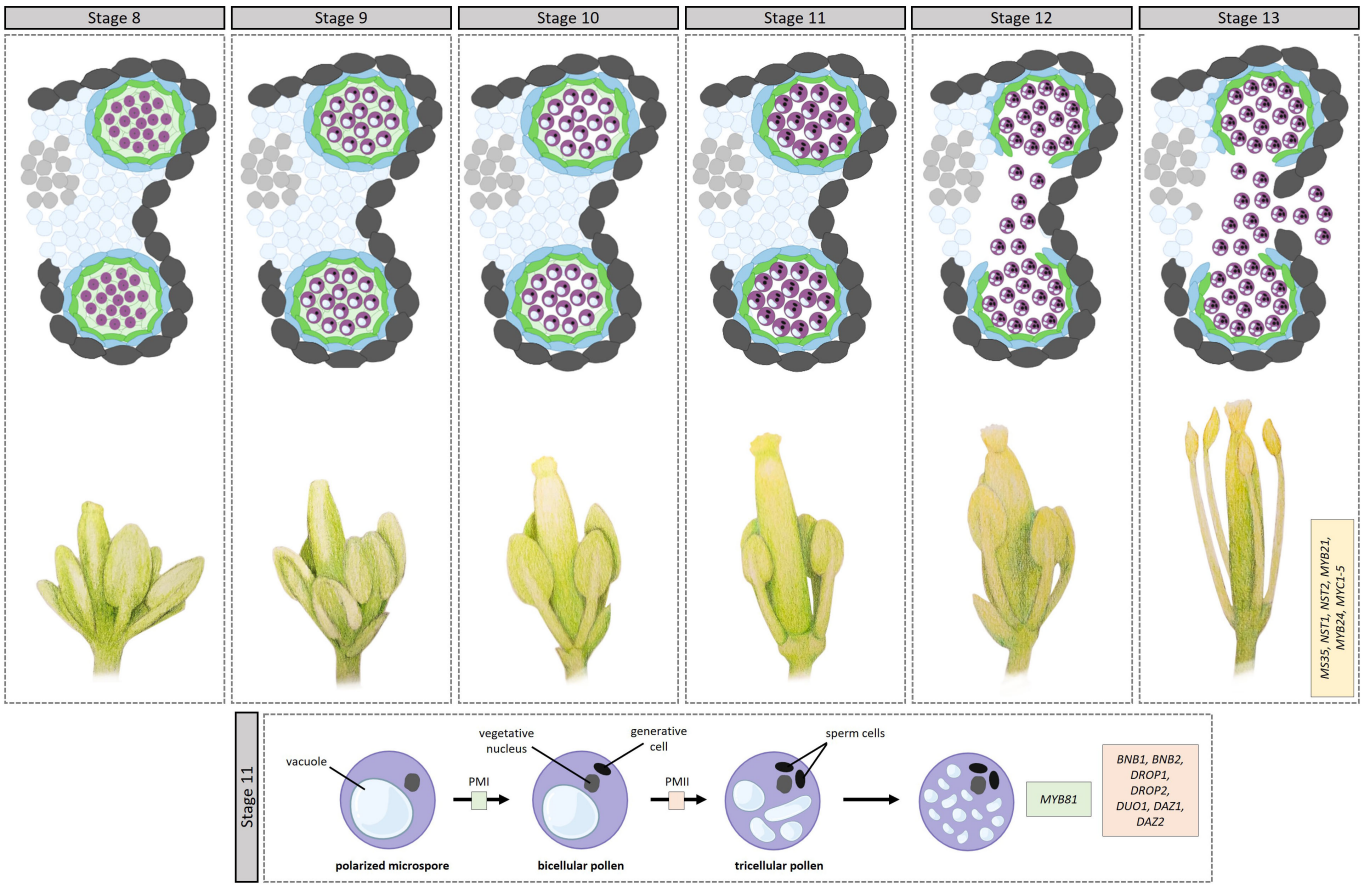
Transcriptional control of microgametogenesis in *Arabidopsis*. Top half op top panel: development through stages 8–13. [8] Microspores, covered by the primexine, are released into the anther locule. [9] Exine formation starts via sporpollenin deposition on the microspores, with the exine eventually becoming thicker and assuming its basic structure. [10] Intine development starts, appearing between the plasma membrane and the nexine. Additionally, the tapetum starts to degrade. [11] This stage comprises two rounds of pollen mitosis. In addition, the septum starts to degrade. [12] The septum breaks, creating a bilocular anther. Pollen are tricullular. [13] Anther dehiscence leads to the anther breaking open along the stomium, releasing mature pollen grains. Bottom half of top panel: anther positioning during stages 8–13. During stages 11–13, the filament elongates substantially, placing the anther in a position to pollinate the stigma. Bottom panel: During pollen mitosis I (PMI), the polarized microspore undergoes a highly asymmetric cell division giving rise to a large vegetative cell and a small generative cell. The smaller generative cell then divides into two sperm cells through PMII. The transcription factors involved in the different steps are indicated in boxes. bofigure in boxes.

During stage 7, after the secretory tapetum has been established, the tapetum synthesizes and secretes callases (β-1,3-glucanase), enzymes that break down the callose wall surrounding the microspores, releasing them from the tetrad into the anther locule. AMS regulates this process through its target gene *MYB80* (Zhang et al., 2006), which plays a role in callose dissolution by regulating the expression of Anther-specific protein 6 (A6), a member of the callase complex. After being expressed and synthesized in the tapetum, UNEVEN PATTERN OF EXINE 1 (UPEX1), encoding an arabinogalactan β-(1,3)-galactosyltransferase, secretes A6 in the anther locule; *UPEX1* expression is directly regulated by AMS (Wang et al., 2022). Following callose dissolution, the microspores, covered by the primexine, are released into the anther locule during stage 8, initiating microgametogenesis (**Figure 3**).

## 4 Microgametogenesis

Once released into the anther locule, the microspores are surrounded by a fluid containing sugars, amino acids, proteins, and sporopollenin precursors secreted by the tapetum, to aid microspore development and pollen wall formation. During stage 9, exine formation continues via sporopollenin deposition on the microspores, with the exine eventually becoming thicker and assuming its basic structure. During microgametogenesis, the formation of the pollen wall starts in earnest, with the ultimate pollen wall comprising an outer exine and an inner intine; reviewed in (Shi et al., 2015). The intine is a pectocellulosic layer synthesized by the microspore, while the exine is composed mostly of sporopollenin synthesized predominantly in the tapetum. The outer exine is further differentiated into an outer sculpted sexine and an inner nexine. The sexine in turn, comprises columns called baculae that support roof-like structures called tecta; in between, the pollen coat (tryphine, also called the pollenkit) is deposited to fill in the spaces of the exine. The pollen coat protects the pollen from dehydration and aids in pollen-stigma adhesion and communication.

MYB80, through its target genes (Wang et al., 2018), is essential for sporopollenin synthesis in the tapetum and ultimately sexine formation; In the *myb80* mutant, the sexine is completely absent (Zhang et al., 2007). Confirmed target genes of MYB80 include: *MALE STERILITY 2* (*MS2*), *POLYKETIDE SYNTHASE A* (*PKSA*) and *PKSB*, and *ACYL-CoA SYNTHETASE5* (*ACOS5*), *TETRAKETIDE α-PYRONE REDUCTASE1* (*TKPR1*) and *TKPR2*, and *CYP703A2*. The proteins encoded by these genes are responsible for catalyzing specific biochemical reactions that modify fatty acids for sporopollenin biosynthesis (Kim et al., 2010; Shi et al., 2015). For example, ACOS5 catalyzes the activation of fatty acids by attaching a Coenzyme A (CoA) molecule to them (de Azevedo Souza et al., 2009). CYP703A2, a CYTOCHROME P450 family protein, catalyzes the hydroxylation of different long chain fatty acids (Morant et al., 2007), which are then either catalyzed into triketide and tetraketide α-pyrones by PKSA and PKSB, or converted to fatty alcohols by MS2 (Aarts et al., 1993). The tetraketide α-pyrones are then likely reduced to polyhydroxylated tetraketide by TKPR1 and TKPR2 (Grienenberger et al., 2010). Outside of the MYB80 regulon, bHLH010 and bHLH089 redundantly contribute to pollen wall formation by regulating genes implicated in the biosynthesis of glyceryl derivatives and flavonols among others (Lai et al., 2022). In addition, basic region leucine zipper 34 (bZIP34) regulates pollen wall development by either directly or indirectly regulating genes involved in lipid metabolism (Gibalova et al., 2009). Later on, bZIP18 was found to be partially functionally redundant with bZIP34, as evidenced by their similar phenotypes and demonstrated interactions; *bzip18* shows pollen abortion, exine formation defects, and the presence of inclusions in the vegetative cell cytoplasm (Gibalová et al., 2017). In addition to these two, a large proportion of other bZIP TFs are expressed in pollen (Honys and Twell, 2004), with interactions among them demonstrated for some (Gibalová et al., 2017). Moreover, bHLH010 and bHLH089 redundantly contribute to pollen wall formation by regulating genes implicated in the biosynthesis of glyceryl derivatives and flavonols among others (Lai et al., 2022).

The sporopollenin precursors are subsequently transported to the anther locule for exine formation by members of the ATP-binding cassette transporter superfamily. AMS regulates the expression of *ABCG26*, the protein product of which transports lipid precursors and polykedtides (Choi et al., 2011; Quilichini et al., 2014). AMS also regulates the expression of *LIPID TRANSFER PROTEIN 12* (*LTP12*) and IMPERFECTIVE EXINE FORMATION (IEF) (Xu et al., 2010). *LTP12* encodes a lipid binding protein that exchanges lipids between membranes while *IEF* encodes a plasma membrane protein that likely transports materials to the anther locule to reinforce sexine and nexine structures (Wang et al., 2021).

During stage 10, intine development also starts, appearing between the plasma membrane and the nexine; here, only the microspores are involved. The NOVEL MICROGAMETOPHYTE DEFECTIVE MUTANT 1 (NMDM1) putative transcription co-activator, regulates intine development by regulating the expression of Arabidogalactin proteins (AGPs) and pectin methylesterases (PMEs) (Mi et al., 2022). Concurrently, the numerous vacuoles in the microspores fuse into a large vacuole, which leads to the migration of the nucleus to one side of the cell, creating a polarized microspore. During stage 10, the tapetum starts degenerating *via* PCD and pollen coat materials start filling in the sexine cavities. MYB80 plays a role in tapetal PCD through its target genes UNDEAD (A1 aspartic protease) and MS1, the penultimate player of the DYT1-TDF1-AMS-MYB80-MS1 module. UNDEAD hydrolyzes apoptosis-inducing proteins in the tapetal mitochondria (Phan et al., 2011) while MS1 regulates the expression of a number of cysteine proteases (Yang C. et al., 2007a). Outside of MYB80 regulation, MYB2 regulates the expression of CEP1 (Guo et al., 2022), a papain-like cysteine protease that directly participates in tapetal PCD (Zhang et al., 2014).

Stage 11 also comprises two rounds of pollen mitosis. During pollen mitosis I (PMI), the polarized microspore undergoes a highly asymmetric cell division giving rise to a large vegetative cell and a small generative cell. The larger vegetative cell is responsible for forming the pollen tube, which will deliver the male gametes to the female reproductive structures during fertilization, while the smaller generative cell will eventually divide into two sperm cells through PMII, which occurs prior to pollen maturation. One TF essential for PMI is MYB81, a microspore-specific TF; *myb81* microspores are unable to undergo PMI, arresting at the polarized microspore stage (Oh et al., 2020). However, the exact mechanisms through which MYB81 promotes microspore progression into PMI remains unclear. Acting later, BONOBO1 (BNB1) and BNB2 redundantly determine the fate of the generative cell after asymmetric microspore division; in the *bnb1bnb2* mutant, the generative cells fail to differentiate into the two sperm cells (Yamaoka et al., 2018). Similarly, DEFECTIVE REGION OF POLLEN 1 (DROP1) and DROP2 are essential for PMII during generative cell division, ensuring the proper formation of the sperm cells; *drop1drop2* mutants lack sperm cells (Zhang et al., 2017). During PMII, DUO POLLEN1 (DUO1) integrates generative cell mitosis and sperm cell differentiation (Brownfield et al., 2009; Borg and Twell, 2011) via a module including its target genes *DUO1-ACTIVATED ZINC FINGER PROTEIN 1* (*DAZ1*) and *DAZ2*, encoding transcriptional repressors, and the corepressor TOPLESS (Borg et al., 2014). Following the completion of pollen mitosis I, and before pollen mitosis II, the exine is visually completed.

During stages 11 and 12, the filament elongates substantially, placing the anther in a position to pollinate the stigma. Here, AUXIN RESPONSE FACTOR 6 (ARF6) and ARF8 play a crucial role in filament elongation, by activating the expression of DEFECTIVE IN ANTHER DEHISCENCE1 (DAD1), a gene associated with jasmonic acid (JA) biosynthesis, which is essential for flower opening and anther dehiscence (Tabata et al., 2010). In the *arf6/arf8* double mutant, a delay in petal and stamen elongation at anthesis is observed, leading to a protrusion of the stigma in unopened flower buds. Moreover, MYB21, a member of the R2R3-MYB transcription factor family, plays a role in regulating flavonol biosynthesis; flavonols, acting as reactive oxygen species scavengers, contribute to stamen development, particularly in filament elongation (Zhang et al., 2021).

During stage 12, tapetum degeneration is mostly complete, releasing their full complement of materials into the anther locule for pollen wall and coat deposition, including flavonoids, alkanes, lipids, proteins, carotenoids (Hsieh and Huang, 2007). The flavonoids specifically, are deposited onto the sporopollenin wall (Jia et al., 2021; Xue et al., 2023). The eventual pollen coat comprises a sticky material that fills in the exine cavities. The pollen coat plays a crucial role in protecting against desiccation and is essential for adhesion, pollen–stigma recognition, and hydration during interactions with the stigma (Jia et al., 2021). The flavonoids in the pollen wall play a crucial role in protecting haploid pollen and spores against environmental stresses like UV-B light, high temperatures, and water loss (Hsieh and Huang, 2007; Xue et al., 2023). During stage 12, the septa break, creating a bilocular anther.

## 5 Anther dehiscence

Stages 13 and 14 comprise anther dehiscence, during which, the anther cells switch from a differentiation to a degeneration program. Anther dehiscence involves lignification of the endothecium cells, degradation of the septum cells to create a “bi-locular anther”, and rupture of the stomium to release the pollen grains at anthesis (Goldberg et al., 1993; Sanders et al., 1999). Most research on male reproductive development has focussed on pollen development, with sparse studies available for anther dehiscence. Nevertheless, some studies have identified TFs implicated in this process. MALE STERILE35 (MS35) is involved in the secondary thickening of the endothecium which is necessary for breaking the stomium, by acting upstream of the lignin biosynthetic pathway (Dawson et al., 1999; Steiner-Lange et al., 2001; Yang et al., 2007b). MS35 also regulates the expression of *NAC SECONDARYWALL THICKENING PROMOTING FACTOR1* (*NST1*) and *NST2*, which act redundantly in regulating secondary cell wall thickening (Mitsuda et al., 2005). The bZIP TFs TGA9 and TGA10 are required for multiple steps in the anther dehiscence program, with mutants showing incomplete dissolution of the middle layer, abnormal lignification of the endothecium, disorganized septums, and nonfunctional stomium-like cells (Murmu et al., 2010). At the intersection of TFs and hormone signaling, MYB21 and MYB24, triggered by jasmonate, play a role in stamen maturation and anther dehiscence (Mandaokar and Browse, 2009). Additional jasmonic acid-regulated TFs include the bHLH TFs MYC1–5, which act redundantly and form transcriptional complexes with MYB21 and MYB24 to control anther dehiscence (Qi et al., 2015). Aside from jasmonic acid, auxin negatively regulates two key events in anther dehiscence, namely endothecium lignification via MYB26 and stomium opening via the control of jasmonic acid (JA) biosynthesis (Cecchetti et al., 2013). Auxin synthesized in anthers, acting through auxin receptors, coordinates anther dehiscence, pollen maturation, and filament elongation; mutants lacking these receptors exhibit premature anther dehiscence (Cecchetti et al., 2008). Moreover, ARF17, is crucial for anther dehiscence by regulating MYB108 expression (Xu et al., 2019), while miR167-mediated downregulation of ARF6 and ARF8 is critical for proper anther growth arrest, enabling anther dehiscence (Zheng et al., 2019).

## 6 From models to crops – capitalizing on conserved pathways and identifying new players

One of the primary benefits of translational research is the identification and manipulation of key TFs and target genes that regulate pollen development in crop plants. By introducing or modifying these TFs, researchers can potentially enhance pollen quality, pollen viability, and pollen production in crop species. In addition, researchers can engineer male sterility in crop plants for hybrid seed production. This prevents self-pollination, facilitating the controlled cross-breeding of male-sterile and fertile lines to create hybrid seeds with desirable traits (e.g., disease resistance or improved yield). This approach offers a significant advantage over manual emasculation and pollination methods, as it streamlines the breeding process and ensures genetic purity. As discussed in the previous sections, misregulation of several pollen- and tapetum-expressed TFs lead to male sterility. Orthologs of these TFs have been identified in several crop species. For example, orthologs of microgametogenesis TFs in rice and maize include those of DYT1 (OsDYT1, ZmMs32), AMS (OsTDR, ZmbHLH51), MYB80 (OsMYB80, ZmMYB84), and MS1 (OsMS1, ZmMs7) ((Wilson and Zhang, 2009). Orthologs of MYB80 have been also been identified in maize, barley, canola, rapeseed, mustard greens, cabbage, and cotton (Xu Y. et al., 2014). Transcriptomic analyses of fertile and sterile lines have identified additional potential TFs that could be manipulated to bring about male sterility. For example. In rapeseed, ca. 250 TFs were differentially expressed between sterile and fertile lines (Jiang et al., 2022). In addition, transcriptomic analyses of thermosenstitive rapeseed lines showed that MADS, NFY, HSF, MYB/C and WRKY TFs may play a role in male fertility under high temperatures (Tang et al., 2019). Aside from male sterility, breeders can enhance additional traits, such as drought or heat tolerance, disease resistance, or nutrient use efficiency in crops by targeting specific TFs. This targeted breeding approach can accelerate the development of improved crop varieties adapted to changing environmental conditions and increasing agricultural challenges.

## 7 Directives for future research

Despite significant progress in the field, much remains to be discovered or understood. For example:

- The regulatory networks governing early anther development and the specification of archesporial cells are not fully elucidated.
- Pollen development is regulated by multiple hormones (e.g., gibberellins, auxins, cytokinins, and jasmonic acid), however, the crosstalk and integration of these hormonal signals with transcriptional regulation remain largely unknown.
- Many TFs undergo post-translational modifications (e.g., phosphorylation, ubiquitination, and acetylation) which can significantly impact their activity and stability. The functional significance of these modifications in pollen development and their precise regulatory roles are still not fully elucidated.
- Pollen development is influenced by various environmental factors (e.g., temperature, light, and nutrient availability), however, how these environmental cues are perceived and transduced into specific transcriptional responses during pollen development requires further investigation.
- Several TFs involved in pollen development belong to gene families, leading to functional redundancy and potential compensation among family members. Understanding the extent of this redundancy and how individual factors contribute to specific aspects of pollen development remains challenging.
- TFs often function by forming complexes and interacting with co-regulators to modulate gene expression. The nature and dynamics of these protein-protein interactions during pollen development are yet to be fully unraveled.

Addressing these open questions will require the integration of various -omics approaches along with functional studies and advanced genetic tools.

## 8 Conclusions

In conclusion, *Arabidopsis* has emerged as a powerful model system for studying the intricate molecular and genetic mechanisms underlying male reproductive development. Throughout this literature review, we have explored the roles of various TFs in the different stages of anther and pollen development, shedding light on their functions in specifying cell fate, regulating pollen wall formation, and ensuring pollen maturation and germination. We have also discussed how the knowledge gained from *Arabidopsis* research on TFs has significant implications for crop improvement. As the field continues to advance, addressing current gaps and open questions will further enrich our understanding of male reproductive development, contributing to the broader goal of sustainable agriculture and global food security.

## Author contributions

AW: Conceptualization, Writing – original draft, Writing – review & editing. ET: Writing – original draft, Writing – review & editing. DH: Funding acquisition, Writing – review & editing.
